# Synthesis and Evaluation of Some Novel Chromone Based Dithiazoles as Antimicrobial Agents

**DOI:** 10.1155/2013/815453

**Published:** 2013-11-27

**Authors:** Naureen Aggarwal, Vishal Sharma, Harpreet Kaur, Mohan Paul Singh Ishar

**Affiliations:** ^1^Bio-Organic and Photochemistry Laboratory, Department of Pharmaceutical Sciences, Guru Nanak Dev University, Amritsar, Punjab 143 005, India; ^2^Department of Microbiology, Guru Nanak Dev University, Amritsar, Punjab 143 005, India

## Abstract

Novel substituted 1,2,4-dithiazolylchromones **3a–j** were synthesized by the reaction of 3-formylchromones (**1a–j**) with two equivalents of *p*-chlorothiobenzamide (**2**) in dry xylene and characterized spectroscopically (IR, ^1^H and ^13^C NMR, mass) and elemental analysis. All synthesized compounds were screened for *in vitro* antimicrobial activity against various pathogenic bacterial and fungal strains and were found to possess good to moderate inhibitory potential against all tested strains. Antimicrobial results reveal that compounds bearing lipophilic electron withdrawing groups such as *chloro* and *bromo* displayed significant inhibitory potential against both bacterial and fungal strains. Particularly, compound **3c** displayed significant inhibitory against bacterial strains and compound **3h** exhibits significant inhibitory potential in comparison to standard drug fluconazole against fungal strain *S. cerevisiae*.

## 1. Introduction

Fungal and bacterial infections are affecting millions of people worldwide, and are associated with high rates of mortality and morbidity [1]. Resistance to antimicrobial agents is becoming a major problem; microbes acquire the ability to resist antimicrobial drugs by undergoing genetic changes either by mutation or gene transfer within or between species that allow microbes to defend themselves against the antimicrobial agents [2]. Therefore, the discovery of new antimicrobials has assumed critical importance to combat the fungal and bacterial infections. Heterocycles containing N and S are found to display variety of biological activities; amongst them, dithiazoles both 1,2,3- and 1,2,4- are endowed with interesting biological activities, in particular, antimicrobial activity [3]. Similarly, chromone moiety constitutes the basic nucleus of flavones, which are most important and widespread natural product of plants and display a large number of biological activities such as antifungal [4], antibacterial [5], anticancer [6], antiviral [7], antioxidant [8] antimalarial [9] neuroprotective [10], and HIV inhibitory [11].

Recently, we have reported the synthesis of 3-(5-phenyl-3H-[1,2,4]-dithiazol-3-yl) chromen-4-ones with significant inhibitory potential against microbial strains; particularly, compound having both electron withdrawing groups such as *chloro* and *fluoro* linked with chromone ring showed more inhibitory potential against fungal strains than standard drug [12]. Therefore, it was decided in the present study to incorporate *chloro* group at the phenyl ring of the 1,2,4-dithiazolylchromones in order to observe the effect of novel substitution on antimicrobial activity.

## 2. Result and Discussion

**Scheme 1:**
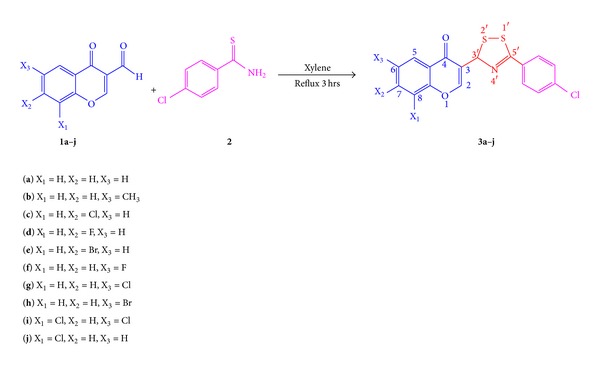
Synthesis of 1,2,4-dithiazolylchromones** 3a–j**.

Substituted 3-formylchromones (**1a–j**) were reacted with two equivalents of *p*-chlorothiobenzamide (**2**) in dry xylene for 3-4 hours leading to the formation of substituted 3-[5′-(*p*-chloro)-phenyl-3*H*-[1,2,4]-dithiazol-3′-yl]-4*H*-chromen-4-one (**3a–j**). The crude mixtures were purified by column chromatography over silica gel (60–120 mesh) and eluted with 1% ethyl acetate in hexane. The purified products were characterized by spectroscopic techniques such as IR, ^1^H and ^13^C NMR, and mass and elemental analysis (Scheme 1, Table 1).

^1^H NMR spectrum of compound** 3c** showed a doublet at *δ* 8.21 (*J* = 0.9 Hz) assigned to C3′-H and another doublet at *δ* 7.75 (*J* = 0.9 Hz) attributed to C2-H of chromone moiety. These NMR spectral features are indicative of an intact chromone ring. ^13^C NMR spectrum showed resonances at *δ* 174.8 (C=O), 156.2 (C3′), 140.3 (C3), and at 82.8 corresponding to (C5′), besides the other anticipated carbon resonances of chromone as well as dithiazole ring of compound **3c**. Its IR spectrum showed a characteristic band at 1645.7 cm^−1^ attributed to (C=O) group and band at 771.4 cm^−1^ corresponding to the disulphide linkage (-S-S-). Mass spectrum revealed a molecular ion peak at m/z 361.5(M^+^) corresponding to molecular mass 361.5 of compound **3c** and its elemental composition is also corroborated by the microanalytical data.

## 3. Antimicrobial Evaluation

### 3.1. Antibacterial Activity

*In vitro* antibacterial activity of compounds (**3a–j**) were established against five human pathogenic bacteria: *Staphylococcus aureus *(MTCC96), *Bacillus subtilis *(MTCC 2451), *Escherichia coli *(MTCC 82), *Pseudomonas aeruginosa *(MTCC 2642), and* Salmonella typhimurium *(MTCC 1251). The antibacterial activity of compounds was determined, with comparison to standard antibiotic discs of amoxicillin and gentamycin. Inhibitory potential of the synthesized compounds was determined in terms of minimum inhibitory concentration (MIC) in *μ*g/mL (Table 2) by using turbidity method.

All synthesized compounds (**3a–j**) were found to be active against all pathogenic bacterial strains. Compound **3c **showed excellent inhibitory activity against *B. subtilis *with MIC 0.78, which is comparable with positive control gentamycin, followed by compound** 3h **with MIC 1.56, wherein compound** 3f **showed inhibitory activity with MIC 3.12. Compounds **3a**, **3c,** and **3e** displayed promising activity against* S. aureus* with MIC 6.25. Compound **3h** exhibited great inhibitory activity against *E. coli* with MIC 1.56 followed by compounds **3c** and **3g **with MIC 3.12, wherein compounds **3f** and **3i **with MIC 6.25. Compounds **3h** and **3c** showed good inhibitory activity against *P. aeruginosa* and *S. typhi* with MIC 6.25 and 12.5, respectively.

### 3.2. Antifungal Activity

*In vitro* antifungal activity of various synthesized compounds (**3a–j**) was carried out against three fungal strains, that is, *Saccharomyces cerevisiae *(MTCC 172), *Candida albicans* (MTCC 3018), and *Cryptococcus gastricus *(MTCC 1715). Fluconazole was used as a positive control. Inhibitory potential of the synthesized compounds was determined in terms of minimum inhibitory concentration (MIC) in *μ*g/mL using turbidity method (Table 3).

In the case of fungal strain *S*.* cerevisiae*, compound **3h** displayed very high inhibitory activity with MIC 0.78, which is more than that of fluconazole used as positive control under similar conditions, followed by compound **3g** with MIC 3.12 and compound **3a** with MIC 6.25. Compound **3c** exhibited promising inhibitory activity against* C*.* albicans* with MIC 3.12, which is higher than standard positive control, followed by compounds **3a**, **3e**, and **3h** with MIC 6.25. Compounds** 3h** showed comparable inhibitory activity against *C*.* gastricus* (MIC 6.25) with standard drug fluconazole, followed by **3c** with MIC 12.5, respectively. The structure activity relationship of all the synthesized compounds was developed on the basis of obtained *in vitro* antimicrobial results. The substitution of -*chloro* group at *para* position of phenyl ring of the 1,2,4-dithiazole enhanced the inhibitory activity against various bacterial and fungal strains comparison to our previously synthesized compounds [12]. It was observed that substitution of electron withdrawing groups at C_6_ and C_7_ positions of chromone ring leads to an increase in both antifungal and antibacterial activities. Therefore, efforts are made to perform substitution with different electron withdrawing groups at C_6_, C_7_, and C_8_ positions of the chromone ring. C_6_ position of chromone moiety also has been substituted with electron donating substitution that is, -methyl, to compare the antimicrobial activity with electron withdrawing substitutions and results revealing that electron donating substitution decreases both antifungal and antibacterial activities. Substitution at C_7_ with electron withdrawing groups *fluoro* and *bromo* leads to an increase in both antifungal and antibacterial activities, especially, against *B. subtilis*, *E. coli*, and *S. cerevisiae*.

## 4. Conclusion

Various substituted 3-[5-(*p*-chloro)-phenyl-3*H*-[1,2,4]-dithiazol-3′-yl]-4*H*-chromen-4-one (**3a–j**) were synthesized and characterized by rigorous spectroscopic techniques such as ^1^H and ^13^C NMR, IR, and mass and elemental analysis. All synthesized compounds (**3a–j**) were screened for *in vitro* antimicrobial activity against various pathogenic bacterial and fungal strains. Compound **3c** exhibited excellent inhibitory activity against *B. subtilis* with MIC 0.78 followed by compound **3h **which displayed promising inhibitory activity against *B. subtilis *and *E. coli *with MIC 1.56. Compound **3h** showed excellent inhibitory activity against *S. cerevisiae* with MIC 0.78 as compared to standard drug fluconazole and compound **3c** showed promising inhibitory activity against *C. albicans* with MIC 3.12. These compounds can be taken as “lead” to develop more potent antimicrobial agents.

## 5. Materials and Methods

### 5.1. Chemistry

Starting materials and reagents were purchased and used after further purification (crystallization/distillation). JEOL AL-300FT (300 MHz) NMR spectrometer was used to record ^1^H and ^13^C NMR (75 MHz) spectra and chemical shifts (*δ*) are reported as downfield displacements from tetramethylsilane (TMS) used as internal standard and coupling constants (*j*) are reported in Hz. IR spectra were recorded with Shimadzu FT-IR-8400S on KBr pellets. Mass spectra (ESI method) were recorded on Bruker Daltonics esquire 300 mass spectrometer and HR-MS (ESI-TOF). Elemental analyses were carried out on a Thermoelectron EA-112 elemental analyser and reported in percent abundance.

### 5.2. Synthesis of Substituted 3-[5′-(p-Chloro)-phenyl-3H-[1,2,4-dithiazol]-3′-yl]-4H-chromen-4-ones (**3a–j**)

The reaction were carried out by refluxing substituted 3-formylchromones (300 mg, 1.30 mmol) with *p*-chlorothiobenzamide (460.2 mg, 3.35 mmol) in a 100 mL round bottom flask using dry xylene (10 mL) as a solvent at a temperature of 200°C. The heating duration was standardized by monitoring the progress of reaction by TLC. After completion of the reaction, the contents were allowed to cool and the solvent (xylene) was evaporated under reduced pressure [12]. The crude mixtures were purified by column chromatography over Silica gel (60–120 mesh, Loba Chemie 20 g, packed in hexane) and eluted with 1% ethyl acetate in hexane. The purified products were characterized with rigorous techniques such as UV, IR, ^1^H and ^13^C NMR, and mass and elemental analysis.

### 5.3. 3-[5′-(p-Chloro)-phenyl-3H-[1,2,4]-dithiazol-3′-yl]-4H-chromen-4-one (**3a**)

Yellow crystalline solid, (140 mg, 40%); mp: 145–148°C;** IR** (CHCl_3_) ***ν***_**max**_ (cm^−1^): 1647, 1465, 761; ^**1**^ **H NMR** (300 MHz, CDCl_3_): *δ* 8.31 (dist. d, 1H, *J* = 8.7 Hz, Ar-H), 8.27 (dist. d, 1H, *J* = 7.5 Hz, Ar-H), 7.90 (d, 1H, *J* = 8.7 Hz, Ar-H), 7.74 (s, 1H, C_5′_H) 7.45 (d, 1H, *J* = 8.4 Hz, Ar-H), 7.25 (s, 1H, C_2_H), 7.21–7.05 (m, 2H, Ar-H); ^**13**^ **C NMR **(75 MHz, CDCl_3_): *δ* 174.8 (C=O), 156.5 (C_5_), 154.2 (q-arom), 152.7 (C_2_), 138.9 (q-arom), 134.1 (q-arom), 130.6 (Ar-CH), 129.4 (Ar-CH), 126.4 (Ar-CH), 125.4 (q-arom), 123.8 (Ar-CH), 122.9 (Ar-CH), 118.4 (Ar-CH), 83.8 (C_3′_); **MS (ESI)** m/z 395.5 (M^+^); **analysis**: Calcad for (C_17_H_10_ClNO_2_S_2_): C 56.74 H 2.80 N 3.89%; found C 56.70 H 2.75 N 3.80%.

### 5.4. 3-[5′-(p-Chloro)-phenyl-3H-[1,2,4]-dithiazol-3′-yl]-6-methyl-4H-chromen-4-one (**3b**)

Yellow crystalline solid, (140 mg, 40%); mp: 155–158°C;** IR** (CHCl_3_) ***ν***_**max**_ (cm^−1^): 1643, 1483, 775; ^**1**^ **H NMR** (300 MHz, CDCl_3_): *δ* 8.30 (d, 1H, *J* = 8.7 Hz, Ar-H), 8.15 (d, 1H, *J* = 8.7 Hz, Ar-H), 8.03 (d, 1H, *J* = 0.9 Hz, C_3′_H), 7.89 (d, 1H, *J* = 8.7 Hz, Ar-H), 7.71 (d, 1H, *J* = 0.9 Hz, C_2_H), 7.48–7.35 (m, 2H, *J* = 8.4 Hz, Ar-H), 1.78 (s. 1H, -CH_3_); ^**13**^ **C NMR **(75 MHz, CDCl_3_): *δ* 174.8 (C=O), 156.5 (C_5′_), 154.7 (q-arom), 152.6 (C_2_), 135.4 (q-arom), 134.1 (Ar-CH), 131.6 (Ar-CH), 129.1 (Ar-CH), 128.8 (Ar-CH), 125.1 (q-arom), 124.8 (q-arom), 122.4 (q-arom), 118.4 (Ar-CH), 83.8 (C_3′_), 22.4 (-CH_3_); **MS (ESI)**: m/z 359.5 (M^+^); **analysis**: Calcad for (C_18_H_12_ClNO_2_S_2_): C 57.82 H 3.24 N 3.75%; found C 57.75 H 3.16 N 3.70%.

### 5.5. 7-Chloro-3-[5′-(p-chloro)-phenyl-3H-[1,2,4]-dithiazol-3′-yl]-4H-chromen-4-one (**3c**)

Yellow crystalline solid, (262 mg, 75%); mp: 181–183°C;** IR** (CHCl_3_) ***ν***_**max**_ (cm^−1^): 1645, 1475, 773; ^**1**^ **H NMR** (300 MHz, CDCl_3_): *δ* 8.30 (d, 1H, *J* = 8.7 Hz, Ar-H), 8.21 (d, 1H, *J* = 0.9 Hz, C_3′_H), 7.98 (d, 1H, *J* = 8.7 Hz, Ar-H), 7.98 (d, 1H, *J* = 8.7 Hz, Ar-H), 7.91–7.87 (m, 2H, Ar-H), 7.75 (d, 1H, *J* = 0.9 Hz, C_2_H); ^**13**^ **C NMR **(75 MHz, CDCl_3_): *δ* 174.8 (C=O), 156.2 (C_5′_), 153.6 (C_2_), 140.3 (q-arom), 138.3 (q-arom), 135.2 (q-arom), 130.5 (Ar-CH), 129.5 (Ar-CH), 128.4 (Ar-CH), 127.5 (Ar-CH), 124.6 (q-arom), 123.4 (q-arom), 118.3 (Ar-CH), 82.8 (C_3′_);** MS (ESI)**: m/z 361.5 (M^+^); **analysis**: Calcad for (C_17_H_9_ClFNO_2_S_2_): C 57.82 H 2.40 N 3.71%; found C 57.73 H 2.32 N 3.65%.

### 5.6. 7-Fluoro-3-[5′-(p-chloro)-phenyl-3H-[1,2,4]-dithiazol-3′-yl]-4H-chromen-4-one (**3d**)

Orange crystalline solid, (187.5 mg, 75%); mp: 180–182°C; **IR** (CHCl_3_) ***ν***_**max**_ (cm^−1^): 1647, 1425, 771; ^**1**^ **H NMR** (300 MHz, CDCl_3_): *δ* 8.24 (d, 1H, *J* = 2.7 Hz, Ar-H), 7.89 (overlapping d, 2H, *J* = 8.4 Hz, Ar-H), 7.88 (d, 1H, *J* = 0.9 Hz C_3′_H), 7.73 (d, 1H, *J* = 0.9 Hz, C_2_H), 7.49–7.41 (m, 2H, Ar-H); ^**13**^ **C NMR **(75 MHz, CDCl_3_): *δ* 174.8 (C=O), 155.3 (C_5′_), 153.2 (q-arom), 152.5 (C_2_), 139.6 (q-arom), 135.4 (Ar-CH), 131.8 (q-arom), 130.5 (Ar-CH), 130.1 (q-arom), 129.7 (Ar-CH), 125.8 (Ar-CH), 123.4 (q-arom), 119.8 (Ar-CH), 82.8 (C_3′_); **HRMS (ESI-TOF)**: Calcad for (C_17_H_9_ClBrNO_2_S_2_): m/z 437.2050; found 437.2050 (M^+^); **analysis**: Calcad for (C_17_H_9_ClBrNO_2_S_2_): C 46.54 H 2.07 N 3.19%; found C 46.46 H 2.02 N 3.12%.

### 5.7. 7-Bromo-3-[5′-(p-chloro)-phenyl-3H-[1,2,4]-dithiazol-3′-yl]-4H-chromen-4-one (**3e**)

Light orange crystalline solid, (200 mg, 80%); mp: 140-143°C; **IR** (CHCl_3_) ***ν***_**max**_ (cm^−1^): 1685, 1442, 771; ^**1**^ **H NMR** (300 MHz, CDCl_3_): *δ* 8.13 (d, 1H, *J* = 8.7 Hz, Ar-H), 8.10 (d, 1H, *J* = 0.9 Hz C_3′_H), 7.92 (d, 1H, *J* = 8.4 Hz, Ar-H), 7.64 (d, 1H, *J* = 0.9 Hz, C_2_H), 7.42–7.31 (m, 2H, Ar-H); ^**13**^ **C NMR **(75 MHz, CDCl_3_): *δ* 174.8 (C=O), 156.6 (C_5′_), 152.6 (C_2_), 140.3 (q-arom), 138.8 (q-arom), 130.5 (Ar-CH), 130.1 (q-arom), 129.3 (Ar-CH), 127.6 (Ar-CH), 126.4 (Ar-CH), 123.2 (q-arom), 122.3 (q-arom), 118.3 (Ar-CH), 82.8 (C_3′_); **HRMS **(**ESI-TOF**): Calcad for (C_17_H_9_Cl_2_NO_2_S_2_) m/z 399.9694; found 399.9694 (M^+^); **analysis**: Calcad for (C_17_H_9_Cl_2_NO_2_S_2_): C 51.78 H 2.30 N 3.55%; found C 51.70 H 2.30 N 3.55%.

### 5.8. 6-Fluoro-3-[5′-(p-chloro)-phenyl-3H-[1,2,4]-dithiazol-3′-yl]-4H-chromen-4-one (**3f**)

Light yellow crystalline solid, (200 mg, 80%), mp: 170–175°C;** IR** (CHCl_3_) ***ν***_**max**_ (cm^−1^): 1649, 1461, 771; ^**1**^ **H NMR** (300 MHz, CDCl_3_): *δ* 8.16 (d, 1H, *J* = 2.4 Hz, Ar-H), 7.92 (d, 1H, *J* = 9.0 Hz, C_2_-H), 7.83 (d, 1H, *J* = 8.7 Hz, Ar-H), 7.65 (d, 1H, *J* = 0.9 Hz C_3′_H), 7.56 (d, 1H, *J* = 9.0 and 2.4 Hz, Ar-H), 7.40 (d, 1H, *J* = 8.7 Hz, Ar-H), 7.35 (d, 1H, *J* = 0.9 Hz, C_2_H); ^**13**^ **C NMR **(75 MHz, CDCl_3_): *δ* 174.8 (C=O), 156.8 (C_5′_), 152.6 (C_2_), 139.1 (q-arom), 134.3 (Ar-CH), 133.8 (q-arom),131.7 (q-arom), 130.5 (Ar-CH), 129.8 (q-arom), 129.3 (C_5_), 125.7 (Ar-CH), 124.8 (q-arom), 119.9 (Ar-CH), 82.8 (C_3′_);** HRMS (ESI-TOF)**: Calcad for (C_17_H_9_Cl_2_NO_2_S_2_) m/z 415.2220; found 415.2220 (M + Na^+^); **analysis**: Calcad for (C_17_H_9_Cl_2_NO_2_S_2_): C 51.78 H 2.30 N 3.55%; found C 51.70 H 2.30 N 3.55%.

### 5.9. 6-Chloro-3-[5′-(p-chloro)-phenyl-3H-[1,2,4]-dithiazol-3′-yl]-4H-chromen-4-one (**3g**)

Orange crystalline solid, (212.5 mg, 85 5%), mp: 183–185°C;** IR** (CHCl_3_) ***ν***_**max**_ (cm^−1^)^:^ 1635, 1458, 775; ^**1**^ **H NMR** (300 MHz, CDCl_3_): *δ* 8.13 (d, 1H, *J* = 8.7 Hz, Ar-H), 7.89 (d, 1H, *J* = 8.7 Hz, Ar-H), 7.69 (d, 1H, *J* = 0.9 Hz C_3′_H), 7.66 (d, 1H, *J* = 1.2 Hz, Ar-H), 7.56 (dd, 1H, *J* = 8.4 and 1.2 Hz, Ar-H), 7.45 (d, 1H, *J* = 8.7 Hz, Ar-H), 7.40 (d, 1H, *J* = 0.9 Hz, C_2_H); ^**13**^ **C NMR **(75 MHz, CDCl_3_): *δ* 174.8 (C=O), 156.3 (q-arom), 153.5 (C_2_), 152.5 (C_5′_), 139.7 (q-arom), 135.6 (Ar-CH), 131.8 (q-arom), 130.5 (Ar-CH), 130.3 (q-arom), 129.3 (Ar-CH), 125.8 (Ar-CH), 123.4 (q-arom), 118.8 (Ar-CH), 82.8 (C_3′_);** HRMS (ESI-TOF)**: Calcad for (C_17_H_9_ClBrNO_2_S_2_) m/z 461.8813; found 461.8813 (M + Na^+^); **analysis**: Calcad for (C_17_H_9_ClBrNO_2_S_2_): C 46.54 H 2.07 N 3.19%; found C 46.46 H 2.02 N 3.12%.

### 5.10. 6-Bromo-3-[5′-(p-chloro)-phenyl-3H-[1,2,4]-dithiazol-3′-yl]-4H-chromen-4-one (**3h**)

Yellow crystalline solid, (200 mg, 80%); mp: 165–169°C;** IR** (CHCl_3_) ***ν***_**max**_ (cm^−1^): 1635, 1458, 775; ^**1**^ **H NMR** (300 MHz, CDCl_3_): *δ* 8.30 (d, 1H, *J* = 8.7 Hz, Ar-H), 7.90 (d, 1H, *J* = 8.7 Hz, Ar-H), 7.71 (d, 1H, *J* = 0.9 Hz C_3′_H), 7.40 (d, 1H, *J* = 0.9 Hz, C_2_H), 7.20 (d, 1H, *J* = 7.8 Hz, Ar-H), 7.16–7.08 (m, 2H, Ar-H); ^**13**^ **C NMR **(75 MHz, CDCl_3_): *δ* 174.8 (C=O), 156.2 (C_5′_), 153.6 (C_2_), 140.3 (q-arom), 138.2 (q-arom), 135.4 (q-arom), 130.5 (Ar-CH), 129.3 (Ar-CH), 128.4 (Ar-CH), 127.5 (Ar-CH), 124.7 (q-arom), 123.4 (q-arom), 118.3 (Ar-CH), 82.8 (C_3′_);** HRMS (ESI-TOF)**: Calcad for (C_17_H_9_ClFNO_2_S_2_) m/z 413.2697; found 413.2697 (M + Na^+^); **analysis**: Calcad for (C_17_H_9_ClFNO_2_S_2_): C 57.82 H 2.40 N 3.71%; found C 57.73 H 2.32 N 3.65%.

### 5.11. 3-[5′-(p-Chloro)-phenyl-3H-[1,2,4]-dithiazol-3′-yl]-6,8-dichloro-4H-chromen-4-one (**3i**)

Orange crystalline solid, (160 mg, 53.3%); mp: 175–178°C;** IR** (CHCl_3_) ***ν***_**max**_ (cm^−1^): 1647, 1442.7, 771; ^**1**^ **H NMR** (300 MHz, CDCl_3_): *δ* 8.39 (d, 1H,*J* = 3.2 Hz, Ar-H), 8.14 (d, 1H, *J* = 1.4 Hz, C_3′_H), 8.07 (d, 1H, *J* = 1.4 Hz, C_2_H), 8.04 (d, 1H, *J* = 3.0 Hz, Ar-H), 7.97–7.26 (m, 2H, Ar-H);^**13**^ **C NMR **(75 MHz, CDCl_3_): *δ* 174.8 (C=O), 156.8 (C_5′_), 156.2 (C_2_), 140.3 (q-arom), 138.5 (q-arom), 130.5 (Ar-CH), 130.1 (q-arom), 129.8 (q-arom), 128.3 (Ar-CH), 126.4 (Ar-CH), 123.5 (q-arom), 122.5 (q-arom), 118.3 (Ar-CH), 82.8 (C_3′_);** analysis**; Calcad for (C_17_H_8_Cl_3_NO_2_S_2_): C 47.62 H 1.88 N 3.27%; found C 47.52 H 1.70 N 3.19%; Calcad for (C_17_H_9_Cl_2_NO_2_S_2_): C 51.78 H 2.30 N 3.55%; found C 51.70 H 2.30 N 3.55%.

### 5.12. 8-Chloro-3-[5′-(p-chloro)-phenyl-3H-[1,2,4]-dithiazol-3′-yl]-4H-chromen-4-one (**3j**)

Yellow crystalline solid, (140 mg, 40%); mp: 171–175°C;** IR** (CHCl_3_) ***ν***_**max**_ (cm^−1^): 1647, 1456, 771; ^**1**^ **H NMR** (300 MHz, CDCl_3_): *δ* 8.19 (dd, 1H, *J* = 8.1 and 1.5 Hz, Ar-H), 7.90 (d, 1H, *J* = 8.4 Hz, Ar-H), 7.87 (d, 1H, *J* = 0.9 Hz C_3′_H), 7.75 (dd, 1H, *J* = 7.5 and 1.5 Hz, Ar-H), 7.48 (d, 1H, *J* = 8.7 Hz, Ar-H), 7.43 (d, 1H, *J* = 0.9 Hz, C_2_H), 7.41-7.36 (m, 1H, Ar-H); ^**13**^ **C NMR **(75 MHz, CDCl_3_): *δ* 174.8 (C=O), 156.8 (C_5′_), 156.2 (C_2_), 139.5 (q-arom), 135.6 (q-arom), 133.6 (q-arom), 131.4 (q-arom), 130.5 (Ar-CH), 129.6 (q-arom), 129.3 (Ar-CH), 125.7 (Ar-CH), 124.8 (q-arom), 119.5 (Ar-CH), 82.8 (C_3′_);** analysis**: Calcad for (C_17_H_9_Cl_2_NO_2_S_2_): C 51.78 H 2.30 N 3.55%; found C 51.70 H 2.30 N 3.55%.

## 6. Biological Activity

Antibacterial and antifungal activities of synthesized compounds were evaluated using the broth macro dilution method to determine the minimum inhibitory concentration (MIC) [13, 14].

### 6.1. Antibacterial Activity

#### 6.1.1. Bacteria and Media

The bacterial strains used were *Staphylococcus aureus *(MTCC 96), *Bacillus subtilis *(MTCC2451)*, Escherichia coli *(MTCC 82), *Pseudomonas aeruginosa *(MTCC 2642), and *Salmonella typhimurium *(MTCC 1251) from Microbial Type Culture Collection, IMTECH Chandigarh, India. Bacteria were cultivated at 37°C in Mueller-Hinton agar medium. The compounds (**3a–j**) were dissolved in methanol and applied in different concentrations. Methanol was used as negative control and amoxicillin and gentamycin were used as positive controls.

#### 6.1.2. MIC Determination

The broth dilution test was performed in test tubes. In twofold serial dilutions of the compounds, a standardised suspension (McFarland turbidity standard) of test bacteria (100 *μ*L) was added to obtain a final concentration. A growth control tube and sterility control tube were used in each test. After overnight incubation at 37°C, the MIC was determined by measuring optical density at 600 nm as the lowest concentration that inhibits growth, evidenced by the absence of turbidity.

### 6.2. Antifungal Activity

#### 6.2.1. Fungi and Media

The fungal strains used were *Saccharomyces cerevisiae *(MTCC 172), *Candida albicans* (MTCC 3018) and *Cryptococcus gastricus *(MTCC 1715). Fungi were cultivated at 26°C in Sabouraud Dextrose Broth (SBD). The compounds (**119a–j**) were dissolved in methanol and applied in different concentrations. Methanol was used as negative control and fluconazole as positive control.

#### 6.2.2. MIC Determination

The broth dilution test was performed in test tubes. The conidial suspension gave the final concentration. A growth tube and sterility control tube were used in each test. After 48 h incubation at 26°C, the MIC was determined visually as the lowest concentration that inhibits growth, evidenced by the absence of turbidity.

## Figures and Tables

**Table 1:** Reaction time (min) and yield (%) of various purified products (**3a–j**).

S. no.	Chromones			Products	Time (h)	Yield (%)
	X_1_	X_2_	X_3_			
(**1**)	H	H	H	**3a**	3	40
(**2**)	H	H	CH_3_	**3b**	3.5	40
(**3**)	H	Cl	H	**3c**	3.5	75
(**4**)	H	F	H	**3d**	3.5	75
(**5**)	H	Br	H	**3e**	3.5	80
(**6**)	H	H	F	**3f**	3.5	80
(**7**)	H	H	Cl	**3g**	3.5	85
(**8**)	H	H	Br	**3h**	3.5	80
(**9**)	Cl	H	Cl	**3i**	3.5	15
(**10**)	Cl	H	H	**3j**	3.5	25

**Table 2:** MIC (*μ*g/mL) of compounds (**3a–j**) against different bacterial strains.

Comp. no.	*B. subtilis *	*S. aureus *	*E. coli *	*P. aeruginosa *	*S. typhi *
**3a**	25	**6**.**25**	50	—	25
**3b**	12.5	50	—	—	—
**3c**	**0**.**78**	6.25	**3**.**12**	25	**12**.**5**
**3d**	**6**.**25**	25	12.5	50	—
**3e**	25	**6**.**25**	25	—	—
**3f**	**3.12**	12.5	**6**.**25**	25	25
**3g**	50	**3**.**12**	**3**.**12**	12.5	50
**3h**	**1**.**56**	12.5	**1**.**56**	6.25	25
**3i**	25	25	**6**.**25**	—	—
**3j**	25	25	—	—	50
Amoxicillin	**0**.**5**	**0**.**5**	**0**.**12**	**1**.**0**	**0**.**9**
Gentamycin	**0**.**75**	**1**.**2**	**0**.**9**	**1**.**8**	**3**.**2**

**Table 3:** MIC (*μ*g/mL) of various compounds (**3a–j**) against different fungal strains.

Comp. no.	*S. cerevisiae *	*C. albicans *	*C. gastricus *	*M. gypseum *
**3a**	25	**6**.**25**	50	75.4
**3b**	25	—	—	>100
**3c**	**6**.**25**	**3**.**12**	**12**.**5**	>100
**3d**	25	25	—	95.2
**3e**	25	**6**.**25**	25	>100
**3f**	**12**.**5**	25	50	>100
**3g**	**3**.**12**	25	50	57.4
**3h**	**0**.**78**	**6**.**25**	**6**.**25**	>100
**3i**	25	50	50	>100
**3j**	50	—	—	70.5
Fluconazole	**1**.**9**	**3**.**9**	**7**.**8**	>100
